# Characterization of Viscoelastic Poisson’s Ratio of Engineering Elastomers via DIC-Based Creep Testing

**DOI:** 10.3390/polym14091837

**Published:** 2022-04-29

**Authors:** Jonathan A. Sotomayor-del-Moral, Juan B. Pascual-Francisco, Orlando Susarrey-Huerta, Cesar D. Resendiz-Calderon, Ezequiel A. Gallardo-Hernández, Leonardo I. Farfan-Cabrera

**Affiliations:** 1 Departamento de Mecatrónica, Universidad Politécnica de Pachuca, Carretera Pachuca-Cd. Sahagún Km. 20, Ex-Hacienda de Santa Barbara, Zempoala 43830, HGO, Mexico; allan16@micorreo.upp.edu.mx (J.A.S.-d.-M.); jbpascualf@hotmail.com (J.B.P.-F.); 2 SEPI-Escuela Superior de Ingeniería Mecánica y Eléctrica, Instituto Politécnico Nacional, Unidad Zacatenco, Col. Lindavista, Ciudad de México 07738, CDMX, Mexico; osusarrey@ipn.mx (O.S.-H.); egallardo@ipn.mx (E.A.G.-H.); 3 Escuela de Ingeniería y Ciencias, Tecnologico de Monterrey, Ave. Eugenio Garza Sada 2501, Monterrey 64849, NL, Mexico; resendiz.cesar@tec.mx

**Keywords:** rubber, material testing, rheology, Poisson’s ratio, viscoelasticity

## Abstract

New data of creep and viscoelastic Poisson’s ratio, ν(t), of five engineering elastomers (Ethylene Propylene-Diene Monomer, Flouroelastomer (Viton^®^), nitrile butadiene rubber, silicone rubber and neoprene/chloroprene rubber) at different stress (200, 400 and 600 kPa) and temperature (25, 50 and 80 °C) are presented. The ν(t) was characterized through an experimental methodological approach based on creep testing (30 min) and strain (axial and transverse) measurements by digital image correlation. Initially, creep behavior in axial and transverse directions was characterized for each elastomer and condition, and then each creep curve was fitted to a four-element creep model to obtain the corresponding functions. The obtained functions were used to estimate ν(t) for prolonged times (300 h) through a convolution equation. Overall, the characterization was achieved for the five elastomers results exhibiting ν(t) increasing with temperature and time from about 0.3 (for short-term loading) to reach and stabilize at about 0.48 (for long-term loading).

## 1. Introduction

Elastomers are viscoelastic materials widely used in engineering applications. The performance of elastomeric mechanical components not only depends on the material’s mechanical properties but also on viscoelastic properties such as creep compliance, stress relaxation and viscoelastic Poisson’s ratio, ν(t), which are time- and temperature-dependent properties. The viscoelasticity of a material is represented by a combination of both elasticity and viscosity properties in different proportions, which is the reason that an elastomer exhibits a variable elastic modulus dependent of time, stress and temperature. The Poisson’s ratio, ν, is defined as the negative constant ratio between transverse and axial strains in a uniaxial state of stress, which is applied along the axial direction for any elastic, homogenous and isotropic material. Practically, for polymers, this property is assumed as a constant, ν, instead of a time and temperature variable, ν(t), in design and performance simulation of engineering components due to the complexity for its experimental determination [1,2,3]. However, the progress of computing and software technology for modern engineering design has promoted the inclusion of more complex or nonlinear properties, namely, the viscoelastic properties (stress relaxation, creep and ν(t)) of soft materials for obtaining more accurate predictions of performance and service life of engineering components via simulation [1,2,3,4]. For elastomers, the data of viscoelastic properties, particularly ν(t), are scarce in the literature. There only few works reporting on the characterization of the time-dependent Poisson’s ratio of some elastomers. For example, Kuggler et al. [4] determined the ν(t) of different Hypalon-based rubbers and hydroxyl terminated polybutadiene rubber by using an optoelectronic system constant strain rate and stress relaxation tests; they found an increase in Poisson’s ratio with time for all cases. Saseendran et al. [5] determined the evolution of ν(t) of the commercial LY5052 epoxy resin at different cure states under uniaxial tension subject to constant deformation stress relaxation testing. They found that Poisson’s ratio evolved from 0.32 to 0.44 over time depending on the cure state of the resin. Pandini and Pegoretti [6] investigated the phenomenology of the dependence of Poisson’s ratio with temperature, time and strain of two crosslinked epoxy resins with different glass transition temperatures using contact extensometers and the simultaneous measurement of the axial and transverse deformations under two dissimilar tensile and relaxation testing. They found that ν(t) increased with strain rate, temperature, and time. Cui et al. [7] proposed a fully numerical framework based on a theory of stress relaxation for the determination of time-dependent Poisson’s ratio for solid propellants (elastomer composites). The time-dependent Poisson’s ratio was obtained under different cohesive parameters, namely, loading conditions (loading temperature, loading rate and fixed strain) and area fraction. They found that the numerical simulation revealed that time-dependent Poisson’s ratio can be nonmonotonic or monotonic depending on different cohesive parameters. In addition, all time-dependent Poisson’s ratios increased at the beginning of the relaxation stage because of cohesive contact. Then, once transverse and axial strains stop changing, all time-dependent Poisson’s ratios achieved equilibrium values. In a more recent research work, Cui et al. [8] proposed constitutive models relating ν(t) with a classical creep constitutive model using a Laplace transform method and compared with stress relaxation models. They found that, in analytical analyses, creep and relaxation models solutions correlated well. It can be a reference that ν(t) can be obtained from creep or stress relaxation data.

According to theory of viscoelasticity [9,10,11], ν(t) can be directly obtained from stress relaxation tests by measuring the transversal strain with time, $\varepsilon_{x}\left(t\right)$, after applying a constant axial deformation, $\varepsilon_{0}$, as expressed by Equation (1).

(1) $$\nu \left(t\right)=-\frac{\varepsilon_{x}\left(t\right)}{\varepsilon_{0}}$$

In this manner, ν(t) is difficult to obtain accurately due to the minimal transverse strain produced with time during a stress relaxation test even using sophisticated measurement technology with high resolution. This is one of the reasons that published data of ν(t) of elastomers are rarely reported. An alternative method to obtain ν(t) is through creep tests followed by a converse methodological approach [9,12]. Creep tests are advantageous because they allow the generation of larger strains in both axial and transverse directions with time under a constant tensile or compressive load, which can be measured more accurately and easily. This methodological approach and its rationalization have been recently published previously elsewhere [13]. It has been demonstrated to be effective for the evaluation of ν(t) of elastomers under different stress levels and temperatures by using digital image correlation (DIC) for strain measurement.

Generally, the measurement of creep strain has been achieved by using gripping extensometers or strain gauges adhered or gripped to the material sample in standard tensile creep testing devices. Nevertheless, the application of these strain measurement gauges can penetrate the sample producing disturbances in structural homogeneity and producing stress concentrators in the material, as well as, restricting the free strain produced in the sample. It is known to introduce some errors in the collected strain data. Hence, in order to avoid errors in the strain measurement, some additional data correction techniques [6] and techniques based on non-contact optical measurement such as Moire interferometry, electronic speckle pattern interferometry (ESPI), shearography, and digital image correlation (DIC) have been applied effectively [12,14,15]. The utilization of a non-contact strain measurement technique such as DIC allows accurate creep strain determination in elastomers without interfering with the sample deformation during the creep strain state. Other alternatives have been proposed and used by several research groups for evaluating the viscoelastic behavior of soft materials such as elastomers, particularly creep. For instance, the standard methods include ASTM-D2990 and ISO 899–1:2003, some non-standard tensile test methods [16], dynamic-mechanical analysis (DMA) [17], some methodologies based on nanoindentation [18,19] and micro- and macro-indentation with axi-symmetric indenters [20,21,22,23]. Nonetheless, DIC-based creep testing, in particular, has been demonstrated as a very suitable and accurate tool for mechanical and viscoelasticity characterization of a wide range of materials, including elastomers [24]. Moreover, DIC has been employed for purposes in elastomers. For example, it has been used for studying fatigue crack growth behavior of elastomers, in which plane strain tensile samples (thin and rectangular strips), also named as pure shear samples, are preferred for this testing [25,26,27].

DIC is a non-invasive optical full-field measurement technique based on the comparison of digital images of an image in different stages of change/deformation. For the comparison of the images, it recognizes patterns with different light intensity of an area. Usually, the light intensity pattern is represented by small and well-defined contrasting points detected in the images taken and processed. The points identified in the undeformed image is recognized by contrasting with the light intensity pattern from the surrounding area. Depending on the light intensity of each point, the points with identical light intensity are identified in the deformed image. About 256 levels of grayscale are used for the digitization of the light intensity in black and white images. Using a single camera-based DIC system is sufficient to measure in-plane (two directions) deformations simultaneously, which is sufficient to determine creep and ν(t).

Thus, the aim of this paper is to obtain and provide new data from an extensive novel characterization of the ν(t) of various common engineering elastomers under different tensile loads and temperatures through creep tests and using a single camera-based DIC for obtaining accurate axial and transverse strain measurements in accordance with the methodology reported in [13], which is described in the following section for purpose of this research.

## 2. Materials and Methods

**Figure 1:**
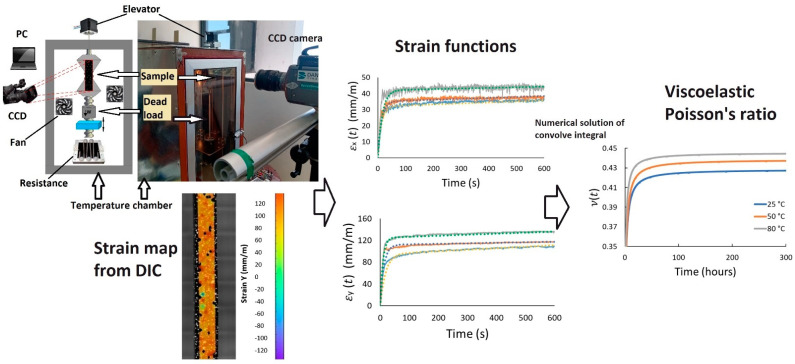
Experimental setup and methodological steps for determining viscoelastic Poisson’s ratio of elastomers.

The ν(t) of five commercial elastomers (Ethylene-Propylene-Diene Monomer (EPDM), Flouroelastomer (Viton^®^) (FKM), nitrile butadiene rubber (NBR), silicone rubber (VMQ) and neoprene/chloroprene rubber (CR)), which are contemporarily used in a wide range of engineering applications (static and dynamic seals, belts, support inserts, vibration insulators, etc.), was determined by a methodological approach using tensile creep tests and strain measurement by DIC. Overall, it comprises the methodological steps shown in Figure 1: (1) measurement of creep strains (generation of the strain map by DIC) in transverse,$\;\varepsilon_{x}\left(t\right)$, and axial, $\varepsilon_{y}\left(t\right)$, directions of an elastomeric sample during a determined creep test period at constant temperature; (2) determination of the creep strain functions in both directions by fitting the obtained data to a known viscoelastic model; (3) estimation of the ν(t) using a numerical solution of a convolution equation for each material and condition.

Both the axial and transverse creep strains of rectangle-shaped samples (60 mm × 5 mm and 2 mm thickness) cut from black sheets of each elastomer were obtained simultaneously by using a DIC equipment (Q-450: Dantec Dynamics, Skovlunde, Denmark) instrumented in a home-built creep test set-up, as shown in Figure 1. Commonly, carbon black is added to the elastomers during their manufacturing process to enhance their mechanical properties [28] and provide black pigmentation to elastomers, which is the case of tested elastomers. It is noteworthy that the strain measurement with DIC can be also applied successfully in the study of materials pigmented with other colors, or even colorless, as long as the required speckle pattern be achieved.

The mechanical properties of the elastomers and parameters of the creep test and DIC measurement are shown in Table 1. The tensile tests were conducted according to the method specified in ASTM-D412 using dumbbell shape specimens with a gauge length of 30 mm at a strain rate of 50 mm/min by using a tensile tester (UE22XX Digital Electronic Tensile Testing Machine, Laryee, Beijing, China) with a load cell of 1 kN. The hardness measurements were performed according to the method in ASTMD2240 in square specimens with dimension of 20 mm using a Shore A type digital durometer (DD-100 Digital Shore Durometer Tester: ABQ Industrial, The Woodlands, TX, USA). The surface roughness of each material was determined in an optical profilometer (Contour GT-K: Bruker, Billerica, MA, USA) with an objective of 5X. The mean roughness of area (Sa) was found to be in the range of 0.2–0.47 µm for all elastomers. The DIC parameters selected have been useful and effective for the creep characterization of elastomers, as reported in a previous research work [24]. The tested samples were finely speckled with white paint for enabling the suitable detection for DIC. The creep tests were run under different proportional tensile stresses and temperatures. They consisted of hanging a rectangular specimen on a frame inside a thermal chamber with temperature control and then applying the predefined tensile load through a dead weight lever system. Once the sample is heated up and maintained at a predefined temperature, the tensile load is applied while DIC measurements are run simultaneously. The sample temperature is measured and monitored by three infrared sensors positioned over different regions of the sample to confirm a quasi-homogenous temperature inside the chamber. To measure $\varepsilon_{x}\left(t\right)$ and $\varepsilon_{y}\left(t\right)$ produced in the sample, a CCD camera (SpeedSense 9070: Phantom, Wayne, NJ, USA) possessing a Zeiss Makro-Planar 50 mm f/2 ZF.2 lens and an image resolution of 1280 × 800 pixels was employed. The camera was connected to a computer loaded with software Istra 4D (Istra 4D: Dantec Dynamics, Skovlunde, Denmark) for the camera configuration, data collection, image processing, and strain computing. The Istra 4D software integrates an algorithm for the numerical calculation of displacements and strain in the x- and y-directions. This calculation is based on the tracking of every point of the image of the speckled sample at any time. Thus, the “true” strain is directly calculated by the software.

The tests were run in triplicate (using new specimens without load history), each for 30 min, for all elastomers and conditions. The time selected was enough to generate the first and second creep stages consistently in all elastomers, which is required to estimate ν(t) [9,12].

Once the creep data ($\varepsilon_{x}\left(t\right)$ and $\varepsilon_{y}\left(t\right)$) were obtained by DIC, the mean creep strain functions ($\varepsilon_{x}\left(t\right)$ and $\varepsilon_{y}\left(t\right)$) were determined from the three repeats. Afterwards, both mean creep functions were used to predict the ν(t) functions for each material and condition by Equation (2), which is a convolution integral equation based on a secure analytical foundation of the viscoelasticity theory [9].

(2) $$\int_{0}^{t}\frac{\nu \left(t-u\right)}{du}\varepsilon_{y}\left(u\right)du=\varepsilon_{x}\left(t\right)+\nu_{g}\varepsilon_{y}\left(t\right)$$

$\nu_{g}$ is the glassy (instantaneous) Poisson’s ratio. Due to there not being an available analytical solution for Equation (1), ν(t) should be evaluated for any time $\left(t_{n}\right)$ using the next recurrence formula [1,12]:

(3) $$\nu \left(t_{n}\right)=\frac{-2\varepsilon_{x}\left(t_{n}\right)+\nu \left(t_{n-1}\right)\left(\varepsilon_{y}\left(t_{0}\right)-\varepsilon_{y}\left(t_{n}-t_{n-1}\right)\right)+X\left(t_{n}\right)}{\varepsilon_{y}\left(t_{n}\right)+\varepsilon_{y}\left(t_{n}-t_{n-1}\right)}$$

where

(4) $$X\left(t_{n}\right)=\sum_{i=1}^{i=n-1}\left(\nu \left(t_{i}\right)+\nu \left(t_{i-1}\right)\right)\left(\varepsilon_{y}\left(t_{n}-t_{i}\right)-\varepsilon_{y}\left(t_{n}-t_{i-1}\right)\right)$$

with

(5) $$\nu \left(t_{0}\right)=\nu_{g}=-\frac{\varepsilon_{x}\left(t_{0}\right)}{\varepsilon_{y}\left(t_{0}\right)}$$

and the following is the case.

(6) $$\nu \left(t_{1}\right)=-\frac{-2\varepsilon_{x}\left(t_{1}\right)+\nu_{g}\left(\varepsilon_{y}\left(t_{1}\right)-\varepsilon_{y}\left(t_{0}\right)\right)}{\varepsilon_{y}\left(t_{1}\right)+\varepsilon_{y}\left(t_{0}\right)}$$

Equation (3) should be evaluated for $t_{n}\geq 2$, where $t_{n}$ is the time to be evaluated, $t_{n-1}$ is the immediate previous time and $t_{0}\;$ stands for t=0. In this manner, ν(t) can be estimated and predicted for the time required.

## 3. Results and Discussions

**Figure 2:**
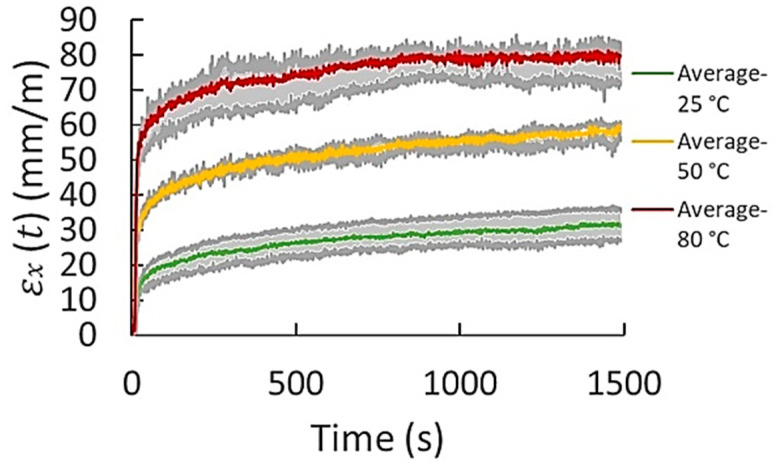
Dispersion of transverse creep data (“x” direction) obtained from three test repeats for FKM at 600 kPa and different temperatures.

**Figure 3:**
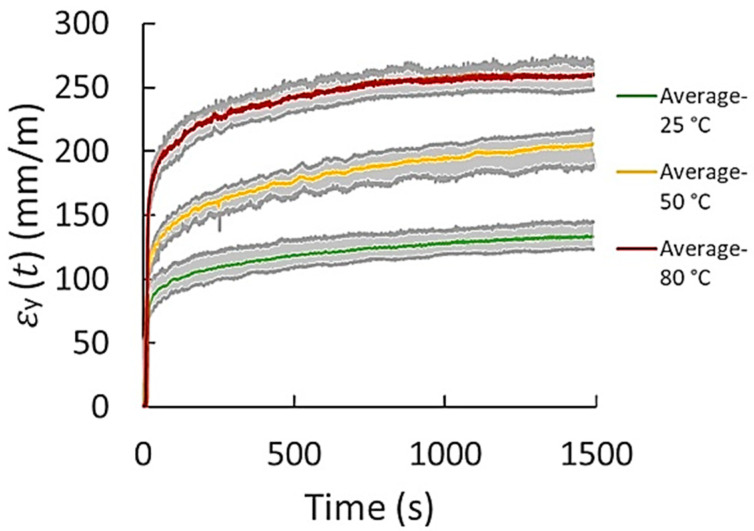
Dispersion of axial creep data (“y” direction) obtained from three test repeats for FKM at 600 kPa and different temperatures.

The data obtained from the three repeats were considered for the creep characterization of all materials and conditions. As an example of the creep raw data results obtained by DIC, the dispersion of the results of transverse and axial creep from the three repeats obtained for FKM at 600 kPa and different temperatures is presented in Figure 2 and Figure 3, respectively. The maximum and minimum creep values, as well as the corresponding average behavior of creep strain of both transverse and axial creep data, are plotted. Considering the dispersion of the three repeats (gray shaded area), an average curve was generated for each case. It was observed that three tests were sufficient to obtain significant repeatability. The standard deviations (% error) obtained from the three repeats were in the range of 1.5–5.1 and 2.2–6.5 mm/m for axial and transverse creep strain, respectively. For most of the materials, it was observed that the higher standard deviations correspond to the measurements of creep in transverse direction (x). This is ascribed to the magnitude of the transverse strains generated; they are very small in contrast to those obtained in the axial direction. The DIC technique is known to be less effective for small strains. When the strain is lower, accuracy and effectiveness become lower. The range of measurement of the DIC system is from 100 micro-strains up to several 100% strain. In addition, other sources causing error in the measurement are rigid body motion when load is applied to the specimen, material structural defects, the non-uniformity in the geometry of the specimens, and the speckle pattern painted over the sample. Rigid body motion is produced when load is applied to the specimen. The electromechanical lever generates small vibrations, especially in the transverse direction, which generates slight oscillations to the specimen. Defects or inconsistencies in the homogeneity, continuity, and properties of the material promote different creep behavior, generating a wider creep strain dispersion. The specimens’ preparation, particularly the cutting of the samples, can produce some irregularities such as non-uniform geometry. Variations in the geometry, in particular the cross-section of the sample, causes higher or less stress to the sample and, therefore, higher or lower strains varying the creep results. On the other hand, in a small extent, another source of the error in the results is the variation of the light intensity captured by the CCD camera together with the non-uniformity of the speckle pattern generated in the surface of the specimens. The ideal speckle pattern for DIC should be composed of many as possible points with similar geometry and light intensity and separated by a well contrasting area in order to an accurate identification of the light patterns by DIC. However, it is very difficult to achieve this speckle pattern homogeneity. Finally, despite the data dispersion obtained by the creep tests and repeats, a clear trend of the creep curves was observed for all materials and test conditions.

For all cases, including all the repeatability tests, the first and second stages of creep were clearly generated in both the axial, $\varepsilon_{x}$, and transverse, $\varepsilon_{y}$, directions. The first stage is characterized by the instantaneous (elastic) strain and an abrupt strain rate decrease while the second creep stage is recognized by a behavior approaching a nearly constant strain rate. Thus, according to the creep strain behavior obtained in both axial and transverse directions for all elastomers and conditions, it was found that all $\varepsilon_{x}$ and $\varepsilon_{y}$ average curves correlated well with a four-element creep model. The model involves the connection in series of the Maxwell and Kelvin–Voigt models [29], as expressed by the following:

(7) $$\varepsilon \left(t\right)=\frac{\sigma_{0}}{R_{1}}+\frac{\sigma_{0}}{\eta_{1}}t+\frac{\sigma_{0}}{R_{2}}\left(1-e^{-\frac{R_{2}t}{\eta_{2}}}\right)$$

where $\sigma_{0}$ is the imposed constant stress, $R_{1}$ and $R_{2}$ are the elastic constants in the Maxwell and Kelvin–Voigt models, respectively, and $\eta_{1}$ and $\eta_{2}$ are the viscous constants in each model. Hence, the data of both mean transverse and axial creep strains were fitted to the model in Equation (7), obtaining the corresponding creep strain function and elastic and viscous constants for each elastomer and condition tested. The average creep functions and the errors (standard deviations) of the fitted models obtained from the three test repetitions conducted for all materials and conditions are summarized in Table 2.

**Figure 4:**
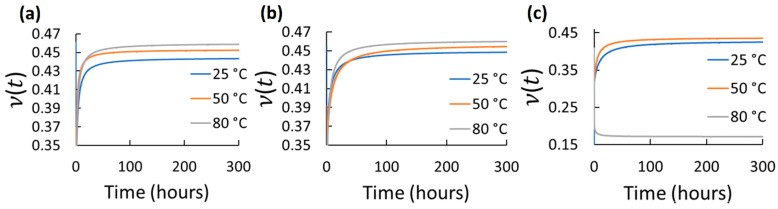
Viscoelastic Poisson’s ratio of EPDM at different temperatures and stress: (**a**) 200 kPa; (**b**) 400 kPa; (**c**) 600 kPa.

**Figure 5:**
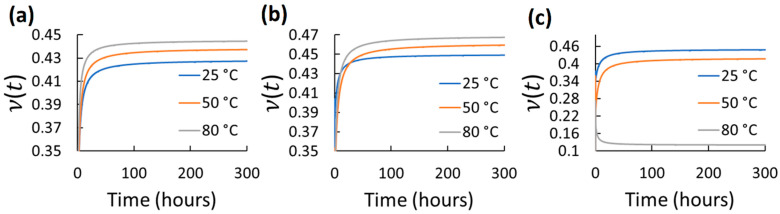
Viscoelastic Poisson’s ratio of CR at different temperatures and stress: (**a**) 200 kPa; (**b**) 400 kPa; (**c**) 600 kPa.

**Figure 6:**
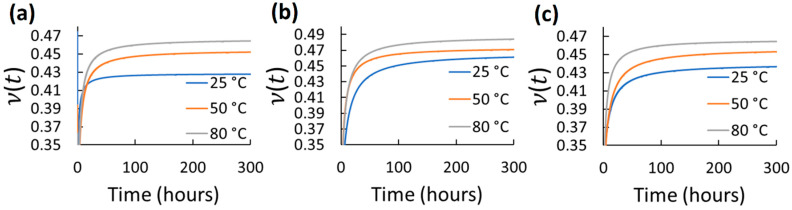
Viscoelastic Poisson’s ratio of NBR at different temperatures and stress: (**a**) 200 kPa; (**b**) 400 kPa; (**c**) 600 kPa.

**Figure 7:**
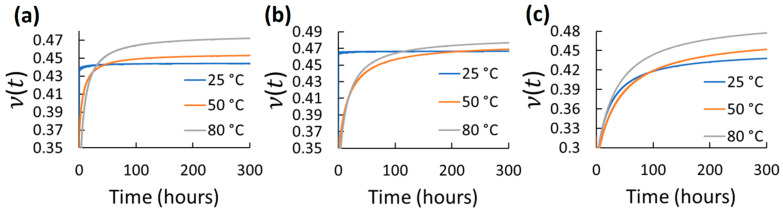
Viscoelastic Poisson’s ratio of VMQ at different temperatures and stress: (**a**) 200 kPa; (**b**) 400 kPa; (**c**) 600 kPa.

**Figure 8:**
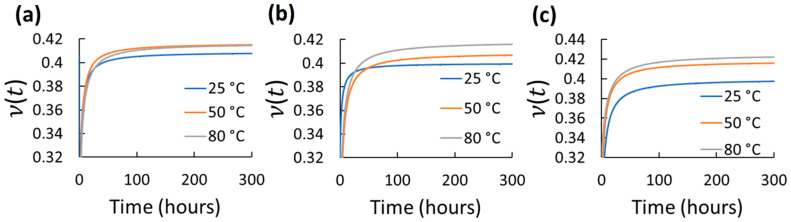
Viscoelastic Poisson’s ratio of FKM at different temperatures and stress: (**a**) 200 kPa; (**b**) 400 kPa; (**c**) 600 kPa.

Using the mean axial and transverse creep functions, ν(t) was determined for 300 h for the different elastomers and test conditions. 300 h were selected as a considerable long-term use period. However, using the same axial and transverse creep function reported in Table 2 and Equations (2)–(6), longer predictions of ν(t) can be estimated. The ν(t) results for each tested elastomer at the different stress and temperature for 300 h are shown in Figure 4, Figure 5, Figure 6, Figure 7 and Figure 8. *t*_0_ was assumed to occur at 1–2 s during stress application since the frame rate employed was 1 fps. Hence, the first strains (used to obtain glassy value) detected by DIC were obtained by the correlation of the two first frames taken. In all cases, except EPDM and CR at the highest temperature (80 °C) and stress (600 kPa), ν(t) increased with time and temperature reaching a stable behavior. The increasing ν(t) with temperature has been demonstrated also for other elastomers, e.g., hydroxyl-terminated poly-butadiene (HTPB) propellant [30]. In the cases of EPDM and CR at the highest temperature and stress, ν(t) decreases with time. It is because these materials are not resistant to those temperatures. They exhibit very high creep rates reaching the third creep stage at high temperatures promoting very large axial strains and minimal transverse strains, which reduces ν(t), especially at high stress. The increase in ν(t)  of the elastomers with temperature is associated to the approach of a liquid-like behavior with increasing temperature, which tends to reach the Poisson’s ratio of an incompressible material (ν≈0.5) [31]. Stress had also influence on ν(t) for all the elastomers; however, it did not exhibit a clear trend. Overall, ν(t) of all the elastomers increased from about 0.3 to 0.48 with time; the last being near to the constant values frequently used for characterizing elastomers (≈ 0.45–0.5). Thus, it can be stated that ν(t) is low (about 0.3) at short-term loading, but it increases and stabilizes to about 0.48 with the long-term loading for the elastomers tested. This growth of *ν*(*t*) with time until reaching an almost stable value has been also reported by other research groups for other viscoelastic materials [4,5,6,30,31,32,33]. It is noteworthy that this behavior is also similar to the ν(t) behavior for stress relaxation for linear viscoelastic materials, as reported in [32,33]. Considering the foundations of linear viscoelasticity, Aili et al. [32] and Charpin and Sanahuja [33] postulated that the instantaneous and the stable *ν*(*t*) are similar for creep and stress relaxation.

Finally, the viscoelastic behaviors of the tested elastomers somehow depend on their molecular weight, crosslinking strength and reinforcements (silica, carbon black, graphene, carbon nanotubes, etc.). However, a deeper analysis of the relationship between chemical composition/structure and *ν*(*t*) is out of the scope of the present work. It requires extensive further research. The new behavior data of ν(t) for different elastomers at different conditions obtained by this characterization work can be useful for modern design of a wide range of elements with different engineering applications in which Poisson’s ratio plays an important role on their performance with short- and long-term use. The experimental method followed in this work for characterizing ν(t)  was demonstrated to be suitable for evaluating different elastomers with relative ease. Moreover, the implementation of DIC for creep measurement allowed an accurate (with acceptable error) measurement of creep without restricting the strain produced in the material opposite to those techniques employing strain gauges gripped on the specimens.

## 4. Conclusions

The viscoelastic properties ($\varepsilon_{x}\left(t\right)$, $\varepsilon_{y}\left(t\right)$ and ν(t)) of ethylene-propylene-diene monomer, flouroelastomer (Viton^®^), nitrile butadiene rubber, silicone rubber and neoprene/chloroprene rubber at different stress (200, 400 and 600 kPa) and temperatures (25, 50 and 80 °C) were successfully characterized through an experimental methodological method based on creep testing and strain measurements via digital image correlation (DIC). The entire field of DIC techniques was successfully employed for the simultaneous measurement of strain in axial ($\varepsilon_{y}$) and transverse ($\varepsilon_{x}$) directions with time. The tests were effective to obtain the first and second creep stages for all elastomers and conditions. The creep behaviors obtained in both the transverse and axial directions for all the materials and conditions were found to correlate well with a four-element creep model. Thus, average creep curves and models for transverse and axial directions for each material and condition were obtained and used for estimating the corresponding ν(t). The reported ν(t) of the five elastomers were estimated through the convolve equation for 300 h. However, it can be determined for more prolonged times by using the axial and transverse creep functions presented in this work and the solution of the convolve equation. Overall, the new data reported in this work suggest ν(t) to increase with temperature and time, raising from about 0.3 (for short-term loading) to reach and stabilize a value to about 0.48 (for long-term loading) for all tested elastomers. Finally, these results can be potentially applied for more accurate analytical or numerical strain and stress analyses of the components made of elastomers for both short- and long-term uses. In addition, the method implemented in this work will facilitate the characterization of complex viscoelastic properties, particularly, creep and ν(t), of conventional and new soft materials (e.g., elastomers), which could be a potential tool for screening materials produced by different manufacturing technologies.

## Figures and Tables

**Table 1:** Material properties and parameters of creep test and DIC measurement.

Material/Test	Property/Parameter	Value
Ethylene-Propylene-Diene Monomer (EPDM) (Manufacturer: Rodillos BMR^®^, Guadalajara, Jalisco, México)	Hardness, ASTM-D2240 (Shore A)	68.5 ± 2
	Tensile breaking strength, ASTM-D412 (MPa)	14 ± 0.6
Flouroelastomer, Viton^®^ (FKM) (Manufacturer: Rodillos BMR^®^, Guadalajara, Jalisco, México)	Hardness, ASTM-D2240 (Shore A)	77.5 ± 2
	Tensile breaking strength, ASTM-D412 (MPa)	11 ± 0.7
Nitrile Butadiene Rubber (NBR) (Manufacturer: Rodillos BMR^®^, Guadalajara, Jalisco, México)	Hardness, ASTM-D2240 (Shore A)	73 ± 2
	Tensile breaking strength, ASTM-D412 (MPa)	6.9 ± 0.5
Silicone rubber/Vinyl-Methyl silicone (VMQ) (Manufacturer: Rodillos BMR^®^, Guadalajara, Jalisco, México)	Hardness, ASTM-D2240 (Shore A)	47.5 ± 1.5
	Tensile breaking strength, ASTM-D412 (MPa)	5 ± 0.8
Neoprene/Chloroprene Rubber (CR) (Manufacturer: Rodillos BMR^®^, Guadalajara, Jalisco, México)	Hardness, ASTM-D2240 (Shore A)	69 ± 2
	Tensile breaking strength, ASTM-D412 (MPa)	3.5 ± 0.5
Strain measurement/DIC parameters	Subset (pixels)	17
	Step (pixels)	3
	Field of view (mm × mm)	55 × 36
	Measurement points (points)	425
	Temporal resolution (fps)	1
	Camera distance (mm)	200
	Image resolution (pixels × pixels)	1280 × 800
	Spatial resolution (mm)	0.1
	Strain resolution (mm/m)	0.25
	Frame amount	1800
	Measurement time (minutes)	30
Creep test	Tensile load (N)	2, 4, 6
	Stress (kPa)	200, 400, 600
	Temperature (°C)	25 ± 1, 50 ± 2 and 80 ± 2
	Test time (minutes)	30

**Table 2:** Axial and transverse creep strain functions for the elastomers and conditions tested.

Material	Stress (kPa)	Temperature (°C)	$\mathrm{Axial}\;\mathrm{Creep}\;\mathrm{Strain}\;\mathrm{Function},\;\varepsilon_{y}\left(t\right)$	Error (%)	$\mathrm{Transverse}\;\mathrm{Creep}\;\mathrm{Strain}\;\mathrm{Function},\;\varepsilon_{x}\left(t\right)$	Error (%)
EPDM	200	25	$\varepsilon_{y}\left(t\right)=20+0.0078t+35\left(1-e^{-0.0492\;t}\right)$	3.7	$\varepsilon_{x}\left(t\right)=5+0.0035\;t+10\left(1-e^{-0.0835t}\right)$	9.5
		50	$\varepsilon_{y}\left(t\right)=30+0.0111\;t+30\left(1-e^{-0.0912\;t}\right)$	5.5	$\varepsilon_{x}\left(t\right)=5+0.0051\;t+13\left(1-e^{-0.0912\;t}\right)$	4.4
		80	$\varepsilon_{y}\left(t\right)=40+0.01754t+85\left(1-e^{-0.0321\;t}\right)$	1.8	$\varepsilon_{x}\left(t\right)=10+0.008\;t+25\left(1-e^{-0.0227\;t}\right)$	3.1
	400	25	$\varepsilon_{y}\left(t\right)=35+0.0069\;t+75\left(1-e^{-0.023\;t}\right)$	1.6	$\varepsilon_{x}\left(t\right)=10+0.0031\;t+28\left(1-e^{-0.0298\;t}\right)$	3.5
		50	$\varepsilon_{y}\left(t\right)=100+0.0061\;t+57\left(1-e^{-0.0121\;t}\right)$	2.0	$\varepsilon_{x}\left(t\right)=35+0.0028\;t+19\left(1-e^{-0.0244\;t}\right)$	2.7
		80	$\varepsilon_{y}\left(t\right)=120+0.0226\;t+120\left(1-e^{-0.0097\;t}\right)$	3.6	$\varepsilon_{x}\left(t\right)=40+0.0104t+30\left(1-e^{-0.0117\;t}\right)$	3.6
	600	25	$\varepsilon_{y}\left(t\right)=150+0.0061\;t+70\left(1-e^{-0.0051\;t}\right)$	3.0	$\varepsilon_{x}\left(t\right)=40+0.0026\;t+30\left(1-e^{-0.0088\;t}\right)$	2.0
		50	$\varepsilon_{y}\left(t\right)=170+0.0139\;t+90\left(1-e^{-0.0062\;t}\right)$	4.0	$\varepsilon_{x}\left(t\right)=60+0.0061\;t+23\left(1-e^{-0.0074\;t}\right)$	3.3
		80	$\varepsilon_{y}\left(t\right)=200+0.0611\;t+350\left(1-e^{-0.0047\;t}\right)$	4.8	$\varepsilon_{x}\left(t\right)=80+0.0104\;t+30\left(1-e^{-0.0055t}\right)$	2.5
CR	200	25	$\varepsilon_{y}\left(t\right)=30+0.0061\;t+18\left(1-e^{-0.0317\;t}\right)$	3.1	$\varepsilon_{x}\left(t\right)=8+0.0026\;t+4\left(1-e^{-0.0212\;t}\right)$	6.3
		50	$\varepsilon_{y}\left(t\right)=40+0.0099\;t+32\left(1-e^{-0.0215\;t}\right)$	1.4	$\varepsilon_{x}\left(t\right)=10+0.0044\;t+7\left(1-e^{-0.0514\;t}\right)$	3.4
		80	$\varepsilon_{y}\left(t\right)=70+0.0192\;t+40\left(1-e^{-0.0204\;t}\right)$	2.4	$\varepsilon_{x}\left(t\right)=15+0.0085\;t+14\left(1-e^{-0.0119\;t}\right)$	4.1
	400	25	$\varepsilon_{y}\left(t\right)=80+0.0069\;t+20\left(1-e^{-0.0084\;t}\right)$	1.9	$\varepsilon_{x}\left(t\right)=10+0.0031\;t+28\left(1-e^{-0.0298\;t}\right)$	4.8
		50	$\varepsilon_{y}\left(t\right)=100+0.0113\;t+54\left(1-e^{-0.0127\;t}\right)$	3.0	$\varepsilon_{x}\left(t\right)=33+0.0052\;t+12\left(1-e^{-0.0218\;t}\right)$	3.8
		80	$\varepsilon_{y}\left(t\right)=140+0.0226\;t+100\left(1-e^{-0.0055\;t}\right)$	2.3	$\varepsilon_{x}\left(t\right)=35+0.0106\;t+35\left(1-e^{-0.01\;t}\right)$	3.2
	600	25	$\varepsilon_{y}\left(t\right)=100+0.0069\;t+56\left(1-e^{-0.0101\;t}\right)$	2.3	$\varepsilon_{x}\left(t\right)=35+0.0031\;t+18\left(1-e^{-0.0147\;t}\right)$	4.6
		50	$\varepsilon_{y}\left(t\right)=170+0.0174\;t+150\left(1-e^{-0.0029\;t}\right)$	2.2	$\varepsilon_{x}\left(t\right)=60+0.0073\;t+12\left(1-e^{-0.0066\;t}\right)$	4.5
		80	$\varepsilon_{y}\left(t\right)=400+0.0874\;t+350\left(1-e^{-0.0031\;t}\right)$	3.1	$\varepsilon_{x}\left(t\right)=100+0.0104\;t+50\left(1-e^{-0.0113\;t}\right)$	4.2
NBR	200	25	$\varepsilon_{y}\left(t\right)=10+0.0036\;t+18\left(1-e^{-0.0660\;t}\right)$	2.0	$\varepsilon_{x}\left(t\right)=2+0.0015\;t+7\left(1-e^{-0.0822\;t}\right)$	11
		50	$\varepsilon_{y}\left(t\right)=25+0.0038\;t+22\left(1-e^{-0.0379\;t}\right)$	3.0	$\varepsilon_{x}\left(t\right)=8+0.0017\;t+3\left(1-e^{-0.1207\;t}\right)$	11
		80	$\varepsilon_{y}\left(t\right)=30+0.0052\;t+22\left(1-e^{-0.0379\;t}\right)$	1.6	$\varepsilon_{x}\left(t\right)=10+0.0024\;t+1\left(1-e^{-0.3615\;t}\right)$	4.6
	400	25	$\varepsilon_{y}\left(t\right)=60+0.0026\;t+15\left(1-e^{-0.024\;t}\right)$	1.0	$\varepsilon_{x}\left(t\right)=10+0.0012\;t+9\left(1-e^{-0.064\;t}\right)$	3.1
		50	$\varepsilon_{y}\left(t\right)=70+0.0066t+23\left(1-e^{-0.0301\;t}\right)$	3.0	$\varepsilon_{x}\left(t\right)=20+0.0031\;t+3\left(1-e^{-0.0572\;t}\right)$	5.5
		80	$\varepsilon_{y}\left(t\right)=90+0.0071\;t+33\left(1-e^{-0.0172\;t}\right)$	2.3	$\varepsilon_{x}\left(t\right)=20+0.0034\;t+10\left(1-e^{-0.036\;t}\right)$	3.6
	600	25	$\varepsilon_{y}\left(t\right)=100+0.0043\;t+33\left(1-e^{-0.0173\;t}\right)$	2.0	$\varepsilon_{x}\left(t\right)=30+0.0019\;t+12\left(1-e^{-0.0145\;t}\right)$	2.4
		50	$\varepsilon_{y}\left(t\right)=120+0.0061\;t+55\left(1-e^{-0.0103\;t}\right)$	2.7	$\varepsilon_{x}\left(t\right)=40+0.0027\;t+12\left(1-e^{-0.007\;t}\right)$	2.6
		80	$\varepsilon_{y}\left(t\right)=150+0.0157\;t+80\left(1-e^{-0.0056\;t}\right)$	2.0	$\varepsilon_{x}\left(t\right)=50+0.0073\;t+17\left(1-e^{-0.0099\;t}\right)$	3.9
VMQ	200	25	$\varepsilon_{y}\left(t\right)=60+0.0015\;t+20\left(1-e^{-0.0232\;t}\right)$	3.2	$\varepsilon_{x}\left(t\right)=10+0.0006\;t+25\left(1-e^{-0.023\;t}\right)$	4.2
		50	$\varepsilon_{y}\left(t\right)=80+0.0034\;t+8\left(1-e^{-0.0578\;t}\right)$	1.9	$\varepsilon_{x}\left(t\right)=20+0.0015t+12\left(1-e^{-0.0479\;t}\right)$	3.6
		80	$\varepsilon_{y}\left(t\right)=80+0.0036\;t+8\left(1-e^{-0.1044\;t}\right)$	1.3	$\varepsilon_{x}\left(t\right)=20+0.0017\;t+5\left(1-e^{-0.0724\;t}\right)$	5.5
	400	25	$\varepsilon_{y}\left(t\right)=140+0.0026\;t+28\left(1-e^{-0.0129t}\right)$	2.6	$\varepsilon_{x}\left(t\right)=40+0.0012\;t+38\left(1-e^{-0.0151\;t}\right)$	3.3
		50	$\varepsilon_{y}\left(t\right)=180+0.0036\;t+45\left(1-e^{-0.0154\;t}\right)$	1.7	$\varepsilon_{x}\left(t\right)=60+0.0017\;t+17\left(1-e^{-0.0102\;t}\right)$	2.4
		80	$\varepsilon_{y}\left(t\right)=200+0.0054\;t+45\left(1-e^{-0.0127t}\right)$	1.5	$\varepsilon_{x}\left(t\right)=70+0.0026\;t+5\left(1-e^{-0.0722\;t}\right)$	2.0
	600	25	$\varepsilon_{y}\left(t\right)=400+0.0069\;t+150\left(1-e^{-0.0115\;t}\right)$	2.0	$\varepsilon_{x}\left(t\right)=120+0.0031t+30\left(1-e^{-0.0278\;t}\right)$	3.0
		50	$\varepsilon_{y}\left(t\right)=400+0.0034\;t+95\left(1-e^{-0.0181\;t}\right)$	1.1	$\varepsilon_{x}\left(t\right)=120+0.0016\;t+17\left(1-e^{-0.0492\;t}\right)$	2.3
		80	$\varepsilon_{y}\left(t\right)=400+0.0034\;t+30\left(1-e^{-0.0154\;t}\right)$	2.2	$\varepsilon_{x}\left(t\right)=100+0.0017\;t+20\left(1-e^{-0.0087\;t}\right)$	2.2
FKM	200	25	$\varepsilon_{y}\left(t\right)=15+0.0038\;t+18\left(1-e^{-0.0256\;t}\right)$	4.3	$\varepsilon_{x}\left(t\right)=3+0.0015\;t+5\left(1-e^{-0.1151\;t}\right)$	22
		50	$\varepsilon_{y}\left(t\right)=40+0.0083\;t+15\left(1-e^{-0.0305\;t}\right)$	2.4	$\varepsilon_{x}\left(t\right)=4+0.0034\;t+5\left(1-e^{-0.0167\;t}\right)$	5.5
		80	$\varepsilon_{y}\left(t\right)=50+0.0062\;t+28\left(1-e^{-0.0203\;t}\right)$	1.4	$\varepsilon_{x}\left(t\right)=10+0.0026\;t+8\left(1-e^{-0.0451\;t}\right)$	5.6
	400	25	$\varepsilon_{y}\left(t\right)=40+0.0052\;t+15\left(1-e^{-0.0114\;t}\right)$	2.4	$\varepsilon_{x}\left(t\right)=10+0.002\;t+8\left(1-e^{-0.0217\;t}\right)$	3.6
		50	$\varepsilon_{y}\left(t\right)=40+0.0076\;t+50\left(1-e^{-0.0166\;t}\right)$	3.8	$\varepsilon_{x}\left(t\right)=10+0.0031\;t+8\left(1-e^{-0.0105\;t}\right)$	3.7
		80	$\varepsilon_{y}\left(t\right)=90+0.0075\;t+45\left(1-e^{-0.0126t}\right)$	2.3	$\varepsilon_{x}\left(t\right)=20+0.0031\;t+15\left(1-e^{-0.024\;t}\right)$	2.8
	600	25	$\varepsilon_{y}\left(t\right)=50+0.0061\;t+55\left(1-e^{-0.0103\;t}\right)$	3.2	$\varepsilon_{x}\left(t\right)=10+0.0024\;t+15\left(1-e^{-0.0115\;t}\right)$	3.9
		50	$\varepsilon_{y}\left(t\right)=80+0.0095\;t+80\left(1-e^{-0.007\;t}\right)$	2.7	$\varepsilon_{x}\left(t\right)=15+0.004\;t+28\left(1-e^{-0.0094\;t}\right)$	2.9
		80	$\varepsilon_{y}\left(t\right)=150+0.0069\;t+60\left(1-e^{-0.0059\;t}\right)$	3.3	$\varepsilon_{x}\left(t\right)=40+0.0029\;t+27\left(1-e^{-0.0064\;t}\right)$	3.1

## Data Availability

Not applicable.
